# History of childhood maltreatment associated with hospitalization or death due to COVID-19: a cohort study

**DOI:** 10.1186/s12916-024-03399-8

**Published:** 2024-08-07

**Authors:** Yue Wang, Fenfen Ge, Thor Aspelund, Helga Ask, Arna Hauksdóttir, Kejia Hu, Jóhanna Jakobsdóttir, Helga Zoega, Qing Shen, Heather C. Whalley, Ole Birger Vesterager Pedersen, Kelli Lehto, Ole A. Andreassen, Fang Fang, Huan Song, Unnur A. Valdimarsdóttir

**Affiliations:** 1 Centre of Public Health Sciences, Faculty of Medicine, University of Iceland, Reykjavík, Iceland; 2 Institute of Environmental Medicine, Karolinska Institutet, Stockholm, Sweden; 3 PsychGen Centre for Genetic Epidemiology and Mental Health, Norwegian Institute of Public Health, Oslo, Norway; 4 Department of Psychology, University of Oslo, Oslo, Norway; 5 School of Population Health, Faculty of Medicine and Health, UNSW Sydney, Sydney, Australia; 6 Clinical Research Center for Mental Disorders, Shanghai Pudong New Area Mental Health Center, Tongji University School of Medicine, Shanghai, China; 7 Institute for Advanced Study, Tongji University, Shanghai, China; 8 Centre for Clinical Brain Sciences, University of Edinburgh, Edinburgh, UK; 9 Generation Scotland, Institute of Genetics and Cancer, University of Edinburgh, Edinburgh, UK; 10 Department of Clinical Medicine, Faculty of Health and Medical Sciences, University of Copenhagen, Copenhagen, Denmark; 11 Department of Clinical Immunology, Zealand University Hospital, Roskilde, Denmark; 12 Estonian Genome Centre, Institute of Genomics, University of Tartu, Tartu, Estonia; 13 Institute of Clinical Medicine, NORMENT Centre, University of Oslo, Oslo, Norway; 14 Division of Mental Health and Addiction, NORMENT Centre, Oslo University Hospital, Oslo, Norway; 15 West China Biomedical Big Data Center, West China Hospital, Sichuan University, Chengdu, Sichuan China; 16 Med-X Center for Informatics, Sichuan University, Chengdu, Sichuan China; 17 Department of Epidemiology, Harvard T H Chan School of Public Health, Boston, MA USA

**Keywords:** COVID-19, Hospitalization, Mortality, Childhood maltreatment, Psychiatric disorders

## Abstract

**Background:**

Childhood maltreatment (CM) has been indicated in adverse health outcomes across the lifespan, including severe infection-related outcomes. Yet, data are scarce on the potential role of CM in severe COVID-19-related outcomes as well as on mechanisms underlying this association.

**Methods:**

We included 151,427 individuals in the UK Biobank who responded to questions on the history of CM in 2016 and 2017 and were alive on January 31, 2020. Binomial logistic regression models were performed to estimate the association between a history of CM and severe COVID-19 outcomes (i.e. hospitalization or death due to COVID-19), as well as COVID-19 diagnosis and vaccination as secondary outcomes. We then explored the potential mediating roles of socio-economic status, lifestyle and pre-pandemic comorbidities, and the effect modification by polygenic risk score for severe COVID-19 outcomes.

**Results:**

The mean age of the study population at the start of the pandemic was 67.7 (SD = 7.72) years, and 56.5% were female. We found the number of CM types was associated with the risk of severe COVID-19 outcomes in a graded manner (*p*_for trend_ < 0.01). Compared to individuals with no history of CM, individuals exposed to any CM were more likely to be hospitalized or die due to COVID-19 (odds ratio [OR] = 1.54 [95%CI 1.31–1.81]), particularly after physical neglect (2.04 [1.57–2.62]). Largely comparable risk patterns were observed across groups of high vs. low genetic risks for severe COVID-19 outcomes (*p*_for difference_ > 0.05). Mediation analysis revealed that 50.9% of the association between CM and severe COVID-19 outcomes was explained by suboptimal socio-economic status, lifestyle, and pre-pandemic diagnosis of psychiatric disorders or other chronic medical conditions. In contrast, any CM exposure was only weakly associated with COVID-19 diagnosis (1.06 [1.01–1.12]) while significantly associated with not being vaccinated for COVID-19 (1.21 [1.13–1.29]).

**Conclusions:**

Our results add to the growing knowledge base indicating the role of childhood maltreatment in negative health outcomes across the lifespan, including severe COVID-19-related outcomes. The identified factors underlying this association represent potential intervention targets for mitigating the harmful effects of childhood maltreatment in COVID-19 and similar future pandemics.

**Supplementary Information:**

The online version contains supplementary material available at 10.1186/s12916-024-03399-8.

## Background

COVID-19 has spread widely around the world and has now resulted in almost 7 million deaths and 100 million hospitalizations worldwide [1, 2]. Both death and hospitalization are commonly used indicators of the severity of COVID-19 illness [3, 4]. Accumulating evidence suggests that older age [5], male sex [5], non-White ethnicity [6], and genetic predisposition [7] are significant risk factors for severe COVID-19. In addition, psychosocial factors such as socio-economic status [8] and pre-pandemic history of psychiatric disorders [9] have been indicated in severe COVID-19 outcomes. While childhood maltreatment is one of the strongest risk factors for both low socioeconomic status [10] and psychiatric disorders in adulthood [11], less is known about its role in COVID-19-related outcomes.

Childhood maltreatment, such as sexual, physical and emotional abuse, or neglect, is common worldwide, affecting 42.2% of children and adolescents in Europe and 58.4% in North America [12]. Previous studies have demonstrated the role of childhood maltreatment in multiple adverse health outcomes across the lifespan, including psychiatric disorders, cardiovascular diseases, cancers, and some infectious diseases [13–17]. Childhood maltreatment might impact COVID-19-related morbidity and mortality outcomes through social, behavioural, emotional, and biological pathways [18, 19]. Indeed, individuals exposed to childhood maltreatment have been reported to have lower socio-economic status (educational attainment and income) in adulthood [10], more likely to have suboptimal health behaviours (e.g. smoking) [20] and immune function [21], and to be at higher risk of obesity [12], cardiovascular disease [22], psychiatric disorders [11], and other chronic diseases in adulthood [13], all of which are associated with severe COVID-19 outcomes [23].

While two previous studies have reported a suggestive link between the history of childhood maltreatment and severe COVID-19 outcomes [24, 25], no study has yet examined the role of specific childhood maltreatment types on severe COVID-19 outcomes nor attempted to disentangle potential underlying mechanisms of this association. Moreover, the role of genetic predisposition to COVID-19-related morbidity and mortality [26] in the association between childhood maltreatment and severe COVID-19-related outcomes remains unknown. Indeed, addressing these knowledge gaps is imperative for health policy and interventions targeting the reduction of potential maltreatment-related negative outcomes in COVID-19 and similar pandemics. Therefore, leveraging the large population-based UK Biobank cohort with pre-pandemic data on childhood maltreatment, we aimed to comprehensively explore the associations between the number and types of childhood maltreatment and severe COVID-19 outcomes, as well as elucidate the mechanisms underlying this association.

## Methods

### Study population and design

We used data from the UK Biobank cohort, which recruited more than 500,000 participants aged 40–69 years from England, Scotland, and Wales between 2006 and 2010. At baseline, participants answered questions on demographic, socio-economic, lifestyle, and health-related factors and provided biological samples for genetic studies [27]. Then, 339,092 participants who agreed to be contacted again were invited to complete online mental health questionnaires during 2016 and 2017, including a retrospective measure of childhood maltreatment. Of the invited participants, 46.4% (*n* = 157,366) responded to this online measure. Despite those respondents being of higher average socio-economic status, the reported mental health problems are comparable to the population prevalence estimates for the corresponding age group [28].

Health-related outcomes (e.g. diagnosis, hospitalization, and death) for the participants were obtained periodically through linkage with multiple national datasets. Specifically, hospital inpatient data were obtained from Hospital Episode Statistics for England (from 1997 to September 30, 2021), the Scottish Morbidity Record for Scotland (from 1981 to July 31, 2021), and the Patient Episode Database for Wales (from 1998 to February 28, 2018). Mortality data were obtained from National Health Service (NHS) Digital for England as well as Wales (from 2006 to September 30, 2021) and NHS Central Register for Scotland (from 2006 to October 31, 2021). Records of COVID-19 test results (by RT-PCR of nose/throat swab samples) were obtained through linkage to Public Health England (i.e. PHE, from March 16, 2020, to September 30, 2021), Public Health Scotland (i.e. PHS, from March 16, 2020, to August 31, 2021), and the Secure Anonymised Information Linkage (SAIL) databank (from March 16, 2020, to August 31, 2021). Additionally, information on the COVID-19 vaccination status was collected for participants of the COVID-19 Self-Test Antibody study (from February 2021 to July 2021). Details of the COVID-19 Self-Test Antibody study are described elsewhere [29, 30].

In the present retrospective cohort study, we included 151,427 participants with information on childhood maltreatment who were alive on January 31, 2020 (i.e. first confirmed COVID-19 cases in the UK) in the analysis. When exploring the potential effect modification by genetic predisposition to severe COVID-19 outcomes, we restricted the analytic sample to only White-British participants considering the variations in genetic predisposition across populations with different ancestries [31, 32]. We excluded participants of non-White British ancestry (*n* = 14,079) or without eligible genotyping data (i.e. genotyping rate < 99%, abnormal heterozygosity level, or kinship coefficient > 0.0884, *n* = 19,555) [33], leaving 117,793 participants in this analysis. Additional file 1: Fig. S1 shows the details of the study profile.

### History of childhood maltreatment

The history of childhood maltreatment was measured using the validated Childhood Trauma Screener (CTS) [34, 35]. It consists of five items assessing whether and how often individuals were exposed to the following types of maltreatment during childhood: sexual abuse, physical neglect, physical abuse, emotional neglect, and emotional abuse, with response options ranging from ‘0’ (never true) to ‘4’ (very often true). Weak correlations were observed among pairs of childhood maltreatment types (Additional file 1: Fig. S2). The questions and threshold values to define each type of childhood maltreatment are based on previous published studies [22, 36] and are shown in Additional file 2: Table S1. In our study, we generated three types of exposure variables: (1) a binary variable indicating any childhood maltreatment, coded as ‘0’ (no) or ‘1’ (yes); (2) a cumulative variable indicating the number of childhood maltreatment types (range from 0 to 5), which was coded as ‘0’, ‘1’, ‘2’, or ‘ ≥ 3’ childhood maltreatment types according to the distribution of the entire study sample (Additional file 1: Fig. S3); and (3) five binary variables for each type of childhood maltreatment, coded as ‘0’ (no) or ‘1’ (yes).

### COVID-19 outcomes

The main outcome of interest was severe COVID-19 outcomes during the study period (i.e. from January 31, 2020, to October 31, 2021). Specifically, participants with a primary diagnosis (i.e. main condition treated or investigated) as COVID-19 (ICD-10, U07.1 or U07.2) in hospital inpatient data or with a cause of death recorded as COVID-19 in death registries were defined as having severe COVID-19 outcomes. A secondary outcome of interest was COVID-19 diagnosis (i.e. tested positive for COVID-19 vs. tested negative for COVID-19), which was determined through records of COVID-19 test results in the PHE, PHS, and SAIL databanks from March 16, 2020, to September 30, 2021. Another secondary outcome of interest was COVID-19 vaccination, based on responses to the question ‘Have you received a first dose of a COVID-19 vaccine?’.

### Genetic predisposition to severe COVID-19 outcomes

We assessed the genetic predisposition to severe COVID-19 outcomes by calculating the polygenic risk score (PRS) for COVID-19 hospitalization or death according to summary statistics (version 5) from the COVID-19 Host Genetics Initiative large-scale GWAS including critically ill COVID-19 patients (*n* = 4,792) and the control population (*n* = 1,054,664) among individuals with European ancestry after excluding UK Biobank and 23andMe participants [32]. We calculated the PRS using the clumping + thresholding approach [37] under 10 *p*-value thresholds (i.e. 5 × 10^−8^, 1 × 10^−7^, 1 × 10^−6^, 1 × 10^−5^, 1 × 10^−4^, 1 × 10^−3^, 0.005, 0.01, 0.05, and 0.1) and validated the association between PRS and severe COVID-19 outcomes in our dataset by fitting logistic regression models adjusting for birth year, sex, genotyping array, and top ten ancestry principal components. We selected the PRS with the highest Nagelkerke *R*^2^ for further analyses (i.e. *p* threshold = 5.00 × 10^−8^; odds ratio = 1.21, 95% CI 1.11–1.32; Nagelkerke *R*^2^ = 2.01%; Additional file 2: Table S2). To avoid inflated type I error from overfitting, we additionally performed a principal component (PC) analysis on the set of the 10 PRSs and used the first PRS-PC for sensitivity analyses [38]. In our dataset, the first PRS-PC for severe COVID-19 outcomes showed a strong association with the severe COVID-19 outcome phenotype (odds ratio = 1.22, 95% CI 1.11–1.33; Nagelkerke *R*^2^ = 2.01%). More information about the PRS-PC analysis is shown in Additional file 1: Fig. S4.

### Covariates

**Fig. 1:**
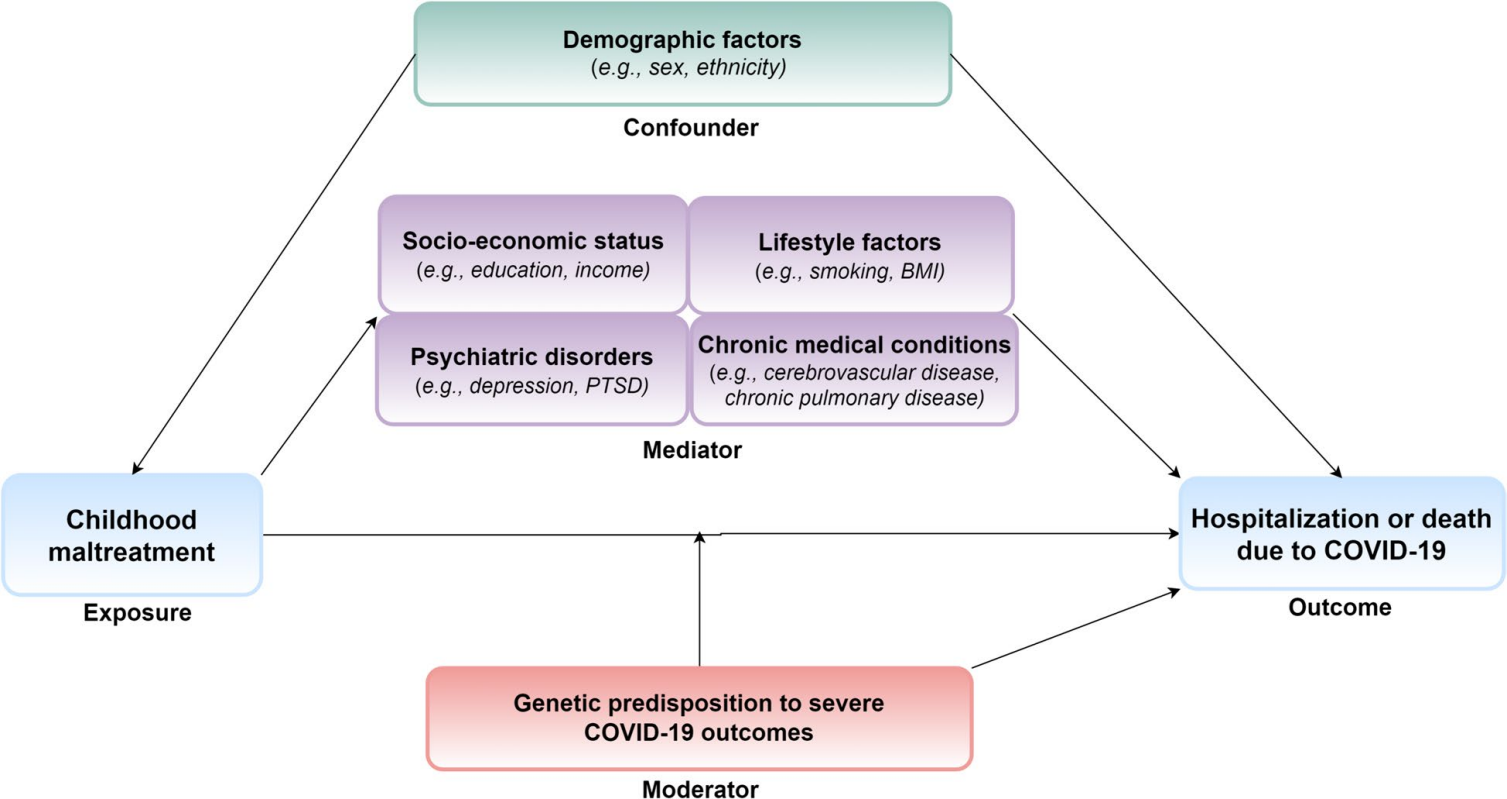
Proposed causal model with alternative pathways of how childhood maltreatment could influence severe COVID-19 outcomes (i.e. hospitalization or death due to COVID-19), taking into account the availability of data

We considered birth year (< 1950, 1950–1959 or ≥ 1960), sex (female or male), ethnicity (White [British, Irish, and any other White background], non-White [mixed, Asian or Asian British, Black or Black British, Chinese, and others], or unknown) and recruitment region (Scotland, England, or Wales) as potential confounders due to previously reported associations to both primary exposure [39, 40] and outcome [5, 6, 41] (Fig. 1). Also, based on previous findings [10–13, 20, 23], we selected four variable clusters as potential mediators: (1) socio-economic status (i.e. Townsend deprivation index [TDI, lower than median, higher than median, or unknown], annual household income [≤ £18,000, £18,000–£30,999, £31,000–£51,999, £52,000–£100,000, ≥ £100,000, or unknown], and college education [no, yes, or unknown]); (2) lifestyle factors (i.e. smoking status [never, previous, current, or unknown] and body mass index [BMI, < 25 kg/m^2^, 25–29.9 kg/m^2^, ≥ 30 kg/m^2^, or unknown]); (3) pre-pandemic chronic medical conditions (no or yes); and (4) pre-pandemic psychiatric disorders (no or yes).

Specifically, TDI was calculated based on the postcode of participants’ address, representing the deprivation at a population level [42]. BMI was calculated using kilogrammes (kg) divided by the square of height in metres (m^2^) using anthropometric data measured at the assessment centre at baseline. We calculated the Charlson Comorbidity Index (CCI) based on Deyo’s coding algorithm [43] using hospital inpatient data (before January 31, 2020), and patients with a CCI ≥ 1 were considered to have pre-pandemic chronic medical conditions. Additional file 2: Table S3 provides more details about the diseases included in the calculation of the CCI. We defined pre-pandemic psychiatric disorders as any diagnosis of psychiatric disorders (ICD-10, F10–F99) in hospital inpatient data before January 31, 2020.

### Statistical analysis

We first compared the characteristics of participants by the history of childhood maltreatment using ANOVAs for continuous data and chi-square tests for categorical data. We then performed binomial logistic regression to estimate the association between a history of childhood maltreatment and severe COVID-19 outcomes, as well as COVID-19 diagnosis and COVID-19 vaccination (i.e. secondary outcomes), with the estimates reported as odds ratios (ORs) and 95% confidence intervals (CIs). The basic model (model 1) was adjusted for demographic factors (i.e. birth year, sex, ethnicity, and recruitment region). In a stepwise approach, the four variable clusters of mediators were additionally adjusted to examine whether and to what extent the ORs between childhood maltreatment and severe COVID-19 outcomes were attenuated (models 2–5). We then conducted a regression-based causal mediation analysis using the CMAverse package in R [44–46] to estimate the proportion of mediation effect by the four variable clusters of mediators individually (M1–M4) and combined (M5). Specifically, the outcomes were regressed by the primary exposure variable (i.e. any childhood maltreatment), specific variable cluster of mediators, and demographic factors in a binomial logistic regression model. Each mediator was then regressed by exposure and demographic factors in either binomial (e.g. pre-pandemic psychiatric disorders) or multinomial (e.g. BMI) logistic regression models. The results of the outcome and mediator models were then combined to calculate the proportion of mediation.

To determine the association of specific types of childhood maltreatment with severe COVID-19 outcomes, we ran separate analyses for each of the five childhood maltreatment types. Furthermore, to examine the potential effect modification by genetic predisposition, we stratified our main analyses of the association between childhood maltreatment (both as a binary variable and a cumulative variable) and severe COVID-19 by tertile of the PRS or the first PRS-PC for severe COVID-19 outcomes (i.e. low, < 1st tertile; intermediate, 1st to 2nd tertile; high, > 2nd tertile). The differences between the groups were tested by introducing interaction terms (i.e. childhood maltreatment × PRS for severe COVID-19 outcomes) in the logistic regression adjusted for birth year, sex, ethnicity, and recruitment region. We then obtained *p*-values to indicate the statistical significance of the interaction terms through the Wald test.

In sensitivity analyses, we first restricted the analysis of the association between childhood maltreatment and severe COVID-19 outcomes to individuals with COVID-19 diagnosis—a population effectively at risk of severe COVID-19-related outcomes. Then, to address the potential impact of COVID-19 vaccination, which started on December 8, 2020, in the UK [47], we reran the main analysis by redefining the study period from January 31, 2020, to December 8, 2020 (i.e. before vaccination roll out). Additionally, given the difference in data coverage across registries (e.g. hospital inpatient data and death registries) and recruitment regions (i.e. England, Scotland, and Wales), we repeated the main analysis by excluding participants registered in Wales as well as by redefining the study period from January 31, 2020, to July 31, 2021. Moreover, instead of using cut-off scores for the measure of childhood maltreatment, we repeated our main analysis using the total CTS score (ranging from 0 to 20), to capture the full range of variability in the severity of childhood maltreatment. Finally, as our primary outcome was hospitalization or death due to COVID-19 as a combined indicator of severe COVID-19, we performed a sensitivity analysis for hospitalization and death due to COVID-19, separately, to determine if there were any differences between these two outcomes.

The regression function for each analysis was shown in the Additional file 3. All analyses were completed using R (version 4.0) and Plink (version 1.9), and a two-tailed test with *p* < 0.05 was considered statistically significant.

## Results

Of 151,427 participants included in the present study, 56.5% were female, and the mean (SD) age at the start of the pandemic was 67.7 (7.72) years. Nearly one-third (*n* = 50,441) of the participants reported at least one type of childhood maltreatment, and emotional neglect (22.2%) was the most commonly reported type, while physical neglect (5.6%) was the least commonly reported type (Additional file 2: Table S1). Compared with unexposed individuals, those who were exposed to childhood maltreatment tended to have a lower level of education and annual household income (*p* < 0.001). They were also more likely to be younger, female, non-White ethnicity, recruited from England, have high BMI (i.e. ≥ 30 kg/m^2^), and with pre-pandemic chronic medical conditions as well as psychiatric disorders (*p* < 0.001; Table 1).

**Table 1:** Characteristics of the study population

	**History of childhood maltreatment**		**Overall (*n* = 151,427)**
	**No (*n* = 100,986)**	**Yes (*n* = 50,441)**	
**Age at the measure of childhood maltreatment**			
Mean (SD)	64.1 (7.69)	63.3 (7.75)	63.8 (7.72)
Median [min, max]	65.0 [46.0, 81.0]	64.0 [46.0, 80.0]	65.0 [46.0, 81.0]
**Age at the start of the pandemic (i.e. 2020)**			
Mean (SD)	68.0 (7.69)	67.2 (7.75)	67.7 (7.72)
Median [min, max]	69.0 [50.0, 84.0]	68.0 [50.0, 84.0]	69.0 [50.0, 84.0]
**Birth year**			
< 1950	44,042 (43.6%)	19,711 (39.1%)	63,753 (42.1%)
1950–1959	36,316 (36.0%)	18,997 (37.7%)	55,313 (36.5%)
≥ 1960	20,628 (20.4%)	11,733 (23.3%)	32,361 (21.4%)
**Sex**			
Female	55,882 (55.3%)	29,709 (58.9%)	85,591 (56.5%)
Male	45,104 (44.7%)	20,732 (41.1%)	65,836 (43.5%)
**Ethnicity**			
White	98,688 (97.7%)	47,975 (95.1%)	146,663 (96.9%)
British	93,211 (92.3%)	44,137 (87.5%)	137,348 (90.7%)
Irish	2044 (2.0%)	1523 (3.0%)	3567 (2.4%)
Others	3433 (3.4%)	2315 (4.6%)	5748 (3.8%)
Non-White	2013 (2.0%)	2250 (4.5%)	4263 (2.8%)
Mixed	356 (0.4%)	428 (0.8%)	784 (0.5%)
Asian or Asian British	650 (0.6%)	595 (1.2%)	1245 (0.8%)
Black or Black British	451 (0.4%)	609 (1.2%)	1060 (0.7%)
Chinese	144 (0.1%)	203 (0.4%)	347 (0.2%)
Others	412 (0.4%)	415 (0.8%)	827 (0.5%)
Unknown	285 (0.3%)	216 (0.4%)	501 (0.3%)
**Recruitment region**			
Scotland	7019 (7.0%)	3137 (6.2%)	10,156 (6.7%)
Wales	3771 (3.7%)	1795 (3.6%)	5566 (3.7%)
England	90,196 (89.3%)	45,509 (90.2%)	135,705 (89.6%)
**Townsend deprivation index**			
Lower than median (< − 2.43)	52,826 (52.3%)	22,825 (45.3%)	75,651 (50.0%)
Higher than median (≥ − 2.43)	48,041 (47.6%)	27,540 (54.6%)	75,581 (49.9%)
Unknown	119 (0.1%)	76 (0.2%)	195 (0.1%)
**College education**			
No	46,757 (46.3%)	24,230 (48.0%)	70,987 (46.9%)
Yes	47,047 (46.6%)	21,806 (43.2%)	68,853 (45.5%)
Unknown	7182 (7.1%)	4405 (8.7%)	11,587 (7.7%)
**Annual household income**			
≤ £18,000	10,924 (10.8%)	7478 (14.8%)	18,402 (12.2%)
£18,000–£30,999	20,838 (20.6%)	10,886 (21.6%)	31,724 (21.0%)
£31,000–£51,999	26,403 (26.1%)	13,223 (26.2%)	39,626 (26.2%)
£52,000-£100,000	24,926 (24.7%)	10,946 (21.7%)	35,872 (23.7%)
≥ £100,000	7812 (7.7%)	3195 (6.3%)	11,007 (7.3%)
Unknown	10,083 (10.0%)	4713 (9.3%)	14,796 (9.8%)
**Smoking status**			
Never	61,041 (60.4%)	26,272 (52.1%)	87,313 (57.7%)
Previous	33,519 (33.2%)	19,440 (38.5%)	52,959 (35.0%)
Current	6215 (6.2%)	4589 (9.1%)	10,804 (7.1%)
Unknown	211 (0.2%)	140 (0.3%)	351 (0.2%)
**Body mass index, kg/m**^**2 a**^			
< 25	40,352 (40.0%)	18,488 (36.7%)	58,840 (38.9%)
25–29.9	41,925 (41.5%)	20,621 (40.9%)	62,546 (41.3%)
≥ 30	18,477 (18.3%)	11,191 (22.2%)	29,668 (19.6%)
Unknown	232 (0.2%)	141 (0.3%)	373 (0.2%)
**Pre-pandemic chronic medical conditions **^**b**^			
No	73,538 (72.8%)	35,050 (69.5%)	108,588 (71.7%)
Yes	27,448 (27.2%)	15,391 (30.5%)	42,839 (28.3%)
**Pre-pandemic psychiatric disorders**			
No	94,307 (93.4%)	44,493 (88.2%)	138,800 (91.7%)
Yes	6679 (6.6%)	5948 (11.8%)	12,627 (8.3%)

^a^The body mass index was calculated using weight kilogrammes (kg) by the square of height in metres (m^2^), using anthropometric data measured at the assessment centre at baseline

^b^We calculated the Charlson Comorbidity Index using hospital inpatient data (before January 31, 2020), and patients with a CCI ≥ 1 were considered to have pre-pandemic chronic medical conditions

**Fig. 2:**
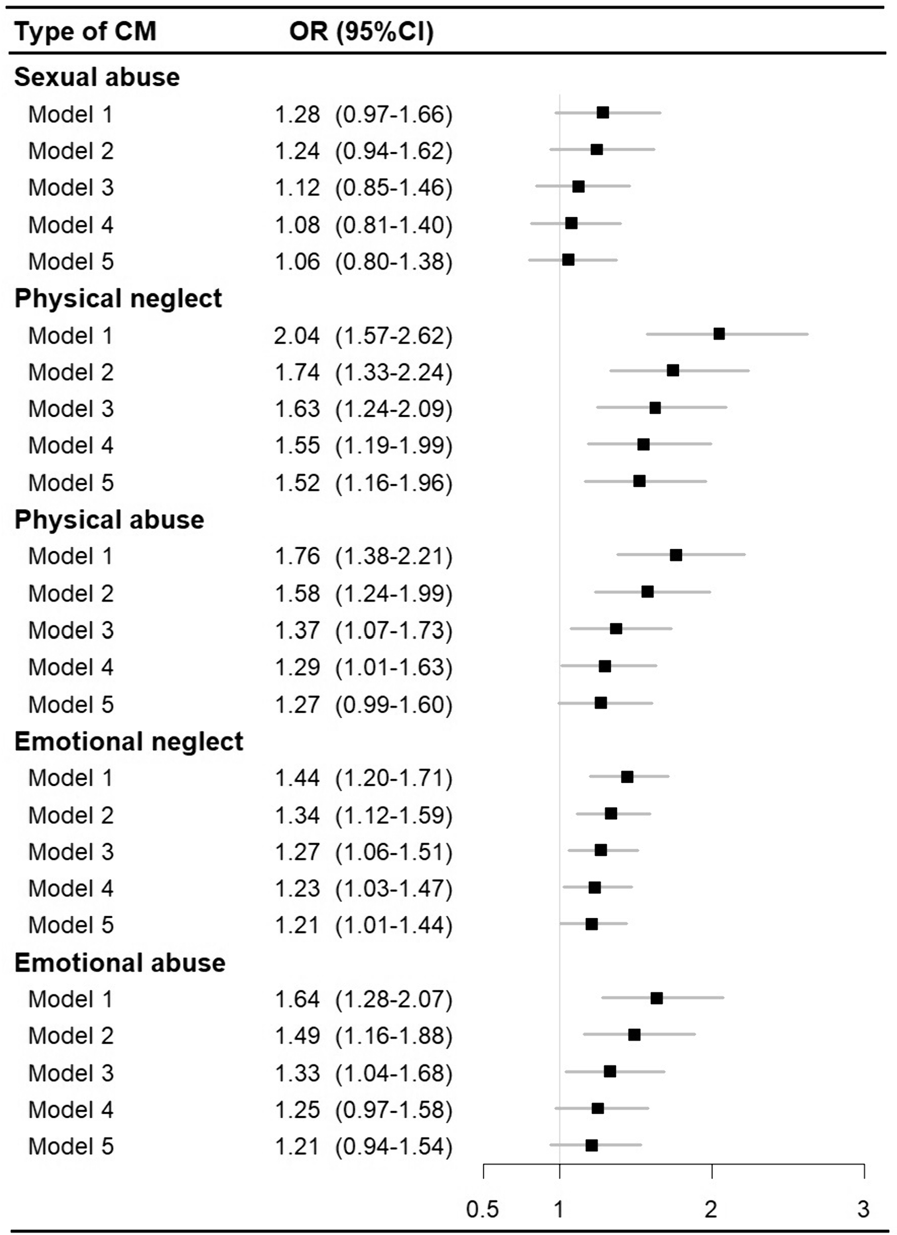
Association between history of childhood maltreatment (CM) and severe COVID-19 outcomes (i.e. hospitalization or death due to COVID-19) by types of childhood maltreatment. *Note*: model 1—adjusted for demographic factors (birth year, sex, ethnicity, and recruitment region); model 2—model 1 additionally adjusted for socio-economic factors (Townsend deprivation index, college education, and annual household income); model 3—model 2 additionally adjusted for lifestyle-related factors (smoking status and body mass index); model 4—model 3 additionally adjusted for pre-pandemic chronic medical conditions (Charlson Comorbidity Index ≥ 1, before January 31, 2020); model 5—model 4 additionally adjusted for pre-pandemic psychiatric disorders (ICD-10, F10–F99; before January 31, 2020)

A total of 606 individuals were hospitalized (*n* = 542) and/or died (*n* = 155) as a result of COVID-19 during the study period. We observed increased odds of severe COVID-19 outcomes among patients exposed to any childhood maltreatment (OR = 1.54 [95% CI 1.31–1.81]; Table 2) when compared with unexposed individuals in the basic model (model 1). The association was amplified in a graded manner by the cumulative number of childhood maltreatment types (*p*_for trend_ < 0.01). Specifically, those who experienced three or more childhood maltreatment types had the highest odds of severe COVID-19 outcomes (2.32 [1.73–3.05]), followed by those who experienced two (1.62 [1.22–2.10]) or one (1.33 [1.09–1.62]) type. The inclusion of potential mediators in the models attenuated the magnitude of the association, although ORs remained statistically significantly higher than one in the fully adjusted model (model 5) among individuals with any childhood maltreatment (1.26 [1.07–1.48]) and those who experienced three or more types of childhood maltreatment (1.50 [1.11–1.98]). Of the five types of childhood maltreatment, physical neglect yielded the strongest association with severe COVID-19 outcomes in the basic model (model 1, 2.04 [1.57–2.62]; Fig. 2) as well as in the model adjusted for all variable clusters of mediators (model 5, 1.52 [1.16–1.96]), although the differences between the groups were not statistically significant (*p*_for difference_ > 0.05).

**Table 2:** Association between history of childhood maltreatment and COVID-19 outcomes (OR and 95% CI)

	**Case/*N* (%)**	**Model 1**^**a**^	**Model 2**^**b**^	**Model 3**^**c**^	**Model 4**^**d**^	**Model 5**^**e**^
**Outcome: severe COVID-19 outcomes (i.e. hospitalization or death due to COVID-19)**						
**Exposure: any childhood maltreatment**						
No	345/100,986 (0.34)	Ref	Ref	Ref	Ref	Ref
Yes	261/50,441 (0.52)	1.54 (1.31–1.81)	1.42 (1.21–1.68)	1.33 (1.12–1.56)	1.28 (1.08–1.51)	1.26 (1.07–1.48)
**Exposure: number of childhood maltreatment types**						
0	345/100,986 (0.34)	Ref	Ref	Ref	Ref	Ref
1	141/30,819 (0.46)	1.33 (1.09–1.62)	1.27 (1.04–1.54)	1.22 (1.00–1.48)	1.20 (0.98–1.46)	1.19 (0.97–1.44)
2	62/11,586 (0.54)	1.62 (1.22–2.10)	1.47 (1.11–1.91)	1.34 (1.01–1.75)	1.29 (0.97–1.68)	1.27 (0.95–1.65)
≥ 3	58/8036 (0.72)	2.32 (1.73–3.05)	2.00 (1.49–2.63)	1.69 (1.26–2.24)	1.56 (1.16–2.06)	1.50 (1.11–1.98)
**Outcome: COVID-19 diagnosis**^**f**^						
**Exposure: any childhood maltreatment**						
No	5362/35,050 (15.30)	Ref	Ref	Ref	Ref	Ref
Yes	2994/18,028 (16.61)	1.06 (1.01–1.12)	1.02 (0.97–1.08)	1.01 (0.96–1.07)	1.02 (0.97–1.07)	1.02 (0.97–1.07)
**Exposure: number of childhood maltreatment types**						
0	5362/35,050 (15.30)	Ref	Ref	Ref	Ref	Ref
1	1735/10,873 (15.96)	1.03 (0.97–1.10)	1.01 (0.95–1.07)	1.00 (0.94–1.06)	1.00 (0.94–1.07)	1.01 (0.95–1.07)
2	714/4209 (16.96)	1.08 (0.99–1.18)	1.04 (0.95–1.13)	1.02 (0.94–1.12)	1.03 (0.94–1.12)	1.03 (0.94–1.13)
≥ 3	545/2946 (18.50)	1.14 (1.03–1.26)	1.07 (0.97–1.18)	1.05 (0.94–1.16)	1.05 (0.95–1.17)	1.06 (0.96–1.18)

^a^Model 1: adjusted for demographic factors (birth year, sex, ethnicity, and recruitment region)

^b^Model 2: model 1 additionally adjusted for socio-economic status (Townsend deprivation index, college education, and annual household income)

^c^Model 3: model 2 additionally adjusted for lifestyle-related factors (smoking status and body mass index)

^d^Model 4: model 3 additionally adjusted for pre-pandemic chronic medical conditions (Charlson Comorbidity Index ≥ 1, before January 31, 2020)

^e^Model 5: model 4 additionally adjusted for pre-pandemic psychiatric disorders (ICD-10, F10–F99; before January 31, 2020)

^f^COVID-19 diagnosis was determined through records of positive COVID-19 test results in the PHE, PHS, and SAIL databanks (*n* = 8356) and compared with individuals who had records of negative COVID-19 test results (*n* = 44,722)

**Fig. 3:**
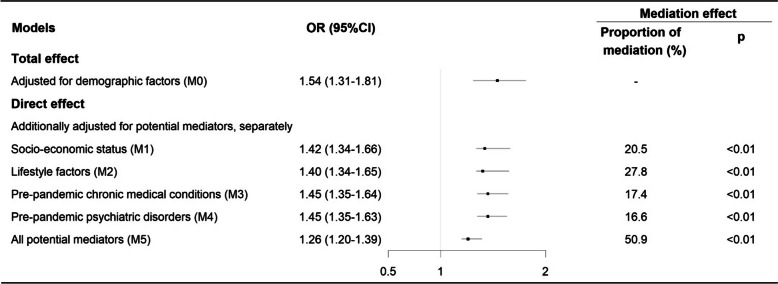
Mediating roles of socioeconomic status, lifestyle, and pre-pandemic chronic medical conditions or psychiatric disorders on the associations between history of childhood maltreatment and severe COVID-19 outcomes (i.e. hospitalization or death due to COVID-19). *Note*: M0—adjusted for demographic factors (birth year, sex, ethnicity, and recruitment region); M1—M0 and additionally adjusted for socio-economic status (Townsend deprivation index, college education, and annual household income); M2—M0 additionally adjusted for lifestyle-related factors (smoking status and body mass index); M3—M0 additionally adjusted for pre-pandemic chronic medical conditions (Charlson Comorbidity Index ≥ 1, before January 31, 2020); M4—M0 additionally adjusted for pre-pandemic psychiatric disorders (ICD-10, F10–F99; before January 31, 2020); M5—M0 additionally adjusted for socioeconomic status, lifestyle, and pre-pandemic chronic medical conditions psychiatric disorders; proportion of mediation: the proportion of the total effect that is mediated through the specified mediators

The majority of the association between childhood maltreatment and severe COVID-19 outcomes was mediated through lifestyle factors (27.8%; Fig. 3), followed by socio-economic factors (20.5%), pre-pandemic chronic medical conditions (17.4%), and psychiatric disorders (16.6%). In total, 50.9% of the association was mediated by all studied mediators and ranged from 49.5% (after physical neglect) to 79.0% (after sexual abuse) across different types of childhood maltreatment.

**Fig. 4:**
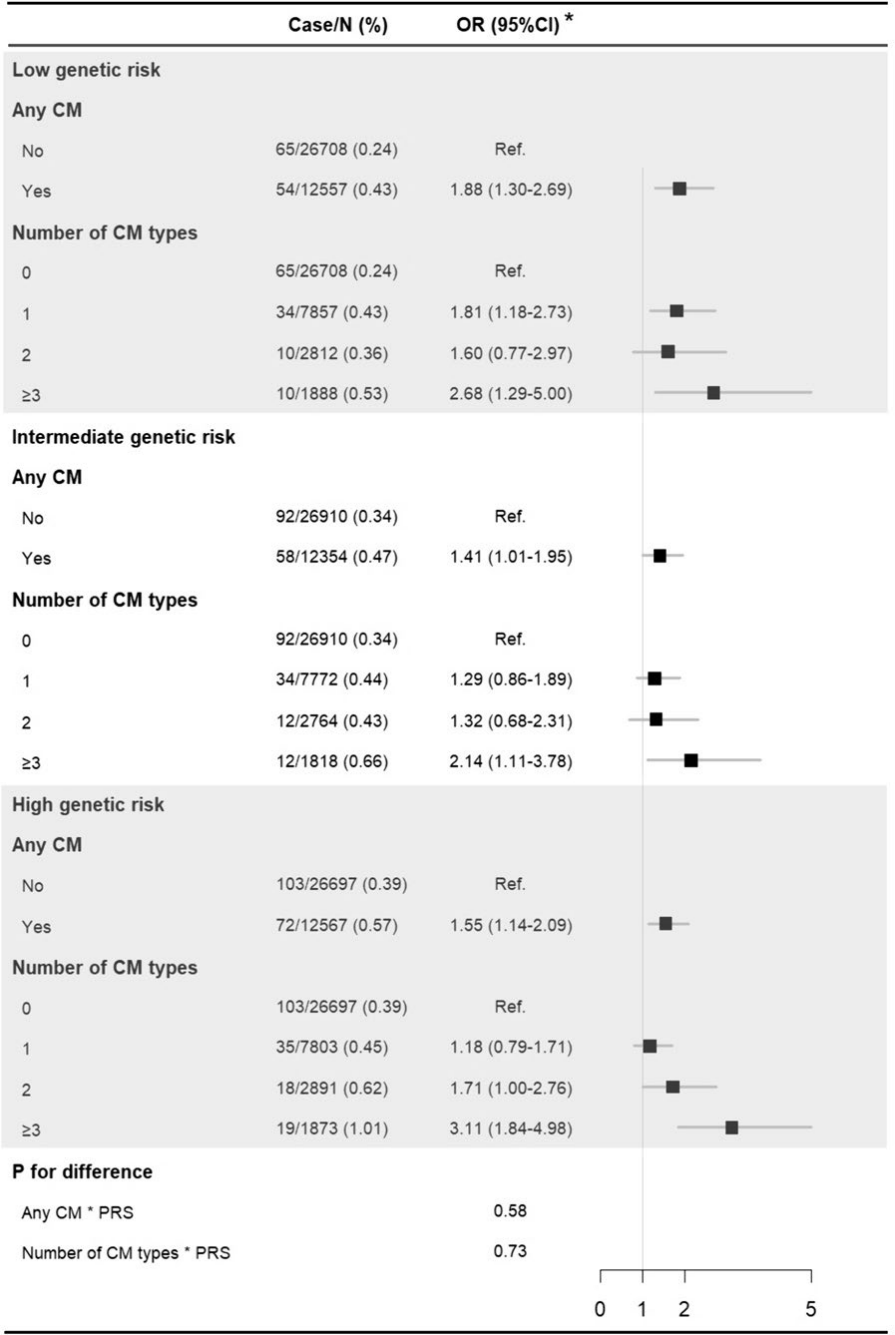
Association between history of childhood maltreatment (CM) and severe COVID-19 outcomes (i.e. hospitalization or death due to COVID-19) by levels of polygenic risk score (PRS) to severe COVID-19 outcomes. *Adjusted for demographic factors (birth year, sex, ethnicity, and recruitment region)

We obtained largely comparable results when stratified by tertiles of PRS for severe COVID-19 outcomes (*p*_for difference_ > 0.05; Fig. 4). Specifically, exposure to any childhood maltreatment (low genetic risk, 1.88 [1.30–2.69]; intermediate genetic risk, 1.41 [1.01–1.95]; high genetic risk, 1.55 [1.14–2.09]) and three or more types of childhood maltreatment (low genetic risk, 2.68 [1.29–5.00]; intermediate genetic risk, 2.14 [1.11–3.78]; high genetic risk, 3.11 [1.84–4.98]) were both consistently associated with significantly increased odds of severe COVID-19 outcomes, regardless of PRS for severe COVID-19 outcomes. We observed similar results when stratifying by the first PRS-PC for severe COVID-19 outcomes (Additional file 1: Fig. S5).

In the sensitivity analyses, we obtained largely comparable results when restricting our analysis to individuals with a COVID-19 diagnosis (Additional file 2: Table S4), redefining the study period before vaccination rollout (Additional file 2: Table S5), and excluding participants registered in Wales and redefining the study period from January 31, 2020, to July 31, 2021 (Additional file 2: Table S6). Also, we observed similar result patterns when using the total CTS score (Additional file 2: Table S7) or analysing hospitalization and death due to COVID-19 as separate outcomes (Additional file 2: Table S8).

Finally, in the secondary analyses, we found any childhood maltreatment and the number of childhood maltreatment types were both consistently associated with significantly increased odds of being unvaccinated for COVID-19 (models 1–5; Additional file 2: Table S9). By contrast, we found a weak association between any childhood maltreatment (model 1, 1.06 [1.01–1.12]) and three or more types of childhood maltreatment (model 1, 1.14 [1.03–1.26]) with COVID-19 diagnosis, which attenuated to null when adding potential mediators to the model (models 2–5; Table 2).

## Discussion

The findings of this cohort study with pre-pandemic data on childhood maltreatment suggest a robust dose–response association between the number of childhood maltreatment types and severe COVID-19 outcomes. While all types of childhood maltreatment were associated with severe COVID-19 outcomes, physical neglect in childhood yielded the strongest association. The associations were partly mediated by suboptimal socio-economic status, lifestyle, and pre-pandemic psychiatric disorders or other chronic medical conditions and were not modified by genetic predisposition to severe COVID-19 outcomes.

In line with the findings of two previous studies [24, 25], our findings confirm the association between childhood maltreatment and severe COVID-19 outcomes. Our findings moreover extend the current level of evidence by showing that all types of childhood maltreatment, ranging from sexual abuse to physical neglect, are robustly associated with severe COVID-19 outcomes. In our study, physical neglect in childhood yielded the highest odds ratios of severe COVID-19-related outcomes, which is similar to the findings from previous studies on other long-term physical outcomes after childhood physical neglect, including test-identified sexually transmitted infections, diabetes, and lung disease [48, 49]. Although the mechanisms underlying this finding remain unclear, it is possible that individuals who experience physical neglect may not receive necessary medical care in childhood, leading to a lack of awareness or appropriate utilization of medical services in adulthood, thereby contributing to the increased risk of severe health consequences in adulthood [50, 51].

Importantly, our findings indicate that more than half of the association between childhood maltreatment and severe COVID-19 outcomes is mediated by suboptimal socio-economic status, lifestyle, and comorbid psychiatric or other chronic medical conditions. These results are consistent with previous findings suggesting that childhood maltreatment may increase the risk of health problems in adulthood through multiple factors, including the adoption of adverse health behaviours and increased vulnerabilities to obesity and other chronic medical conditions of relevance for COVID-19 severity [13, 20, 23]. In line with previous findings [52, 53], we found that childhood maltreatment survivors were more likely to be unvaccinated against COVID-19 which may result in greater risks of severe COVID-19 outcomes [54]. Yet, in the present study, most of the severe COVID-19 outcome events occurred before the introduction of the COVID-19 vaccine, and therefore, we observed similar estimates when redefining the study period before the vaccination rollout.

We further found the history of psychiatric disorders to mediate the association between childhood maltreatment and severe COVID-19 outcomes. Indeed, there is strong evidence for the associations between childhood maltreatment and the risk of psychiatric disorders [11], coupled with our [9] and more recent findings [55] indicating a role of pre-existing psychiatric disorders in severe COVID-19 outcomes. Among the four studied variable clusters of mediators, lifestyle-related factors appear to have the strongest contribution to the association between childhood maltreatment and severe COVID-19 outcomes. However, there is an established link between lifestyle factors and socio-economic status [56], as well as multiple diseases, including cardiometabolic conditions [57] and mental disorders [58]. Therefore, the proportion mediated by each cluster of mediators, as suggested in the causal mediation analysis, is likely confounded by the other mediating clusters.

We found that the association between childhood maltreatment and severe COVID-19 outcomes remained robust after controlling for these potential mediators as well as genetic susceptibility to severe COVID-19 outcomes. Therefore, other unmeasured biological pathways, including disruption of inflammatory responses [59] and hormonal dysregulation [19], may contribute to the elevated risk of severe COVID-19 outcomes. For instance, childhood maltreatment has been associated with immune dysregulation [60, 61], such as disruption in immune cell activation [62], increased proinflammatory cytokine production [63], and accelerated telomere erosion [64], which may reduce an individual’s capacity to recover from COVID-19. Indeed, recent evidence suggests that elevated IL-6 and TNF-α levels can predict disease severity and survival in patients with COVID-19 [65]. In addition, previous studies report an atypical hypothalamic–pituitary–adrenal axis stress response among childhood maltreatment survivors [66], which has been identified as a potential risk factor of severe illness in COVID-19 [67]. In contrast, increased susceptibility to COVID-19 infection is an unlikely explanation for the elevated risk of severe COVID-19-related outcomes by childhood maltreatment, as we found weak or no associations between childhood maltreatment and COVID-19 diagnosis.

### Strengths and limitations of this study

The major strength of our study is the use of a longitudinal study design, i.e. pre-pandemic individual data on childhood maltreatment and follow-up data on COVID-19, in a large population-based cohort. This ensures that the measures of childhood maltreatment indeed preceded any severe COVID-19 outcomes and hence minimizes the risk of reverse causality. Additionally, the primary outcome of interest was death or hospitalization with COVID-19 as the primary diagnosis, as opposed to also including secondary diagnoses in a previous study [25], reducing risks of misclassification of the outcome. Also, by utilizing severe COVID-19 events as the outcome, the influence of surveillance bias should be minor. Moreover, our consideration of genetic predisposition to severe COVID-19 outcomes and a wide range of mediators provides evidence of pathways linking childhood adversities to severe COVID-19 outcomes, with potential relevance for prevention and intervention strategies.

This study also has several limitations to be noted. First, as in most studies on childhood maltreatment, information on childhood maltreatment was recalled by participants in middle or older age rather than captured prospectively (in childhood), which may be liable to underreport [28] and biased by current mental state [68]. However, to explain the observed result pattern, such measurement error would have to be systematic in relation to later severe COVID-19 outcomes. Second, we do not have information on childhood poverty or parental socio-economic status, and several included mediators (e.g. smoking status, BMI) were only measured once at baseline and might have changed over the 10-year follow-up. Third, the incidence of COVID-19 varied across populations and geographical regions [41], yet our sensitivity analyses restricted to individuals with a COVID-19 diagnosis, excluding participants registered in Wales and confined to the study period from January 31, 2020, to July 31, 2021, suggested a minimal influence of these factors on the reported associations. Fourth, the identification of COVID-19 cases relies solely on RT-PCR testing which may lead to underestimation of the COVID-19 diagnosis. Also, the identified hospitalization or mortality rate in our study is lower than the reported rate in the UK during the same period [2]. Indeed, there is evidence of a ‘healthy volunteer’ selection bias of the UK participants who were more likely to live in less socioeconomically deprived areas and have lower rates of all-cause mortality [69]. In addition, most severe childhood maltreatment cases were probably not included in the cohort, possibly resulting in an underestimation of the studied association. Finally, the UK Biobank cohort is not representative of the entire UK population [69], and only approximately 30% of the UK Biobank participants were included in our analysis; thus, the generalization of our findings should be made with caution.

## Conclusions

Our findings suggest that a history of childhood maltreatment, including exposure to physical and emotional neglect or abuse, is robustly associated with severe COVID-19 outcomes. This association was not modified by genetic predisposition to severe COVID-19 outcomes but partly mediated by suboptimal socio-economic status, lifestyle factors, and comorbidities. The latter constitute potential targets for clinical and public health interventions. These findings highlight the role of early life adversities in severe health consequences across the lifespan and call for increased clinical surveillance of people exposed to childhood maltreatment in COVID-19 outbreaks and future pandemics.

### Supplementary Information

**Additional file 1: Fig. S1.** Study profile. **Fig. S2.** Spearman rank correlation matrix for different types of childhood maltreatment. **Fig. S3.** Distribution of cumulative number of childhood maltreatment types. **Fig. S4.** Principal component on the set of the 10 polygenic risk score for severe COVID-19 outcomes. **Fig. S5.** Association between history of childhood maltreatment and severe COVID-19 outcomes, by levels of first PRS-PC to severe COVID-19 outcomes.

**Additional file 2: Table S1.** Participants’ response to the 5 types of childhood maltreatment. **Table S2.** Associations between polygenic risk scores for severe COVID-19 outcomes. **Table S3.** Diseases used for calculating Charlson comorbidity index. **Table S4.** Association between history of childhood maltreatment and severe COVID-19 outcomes, restricted analysis to individuals with COVID-19 diagnosis. **Table S5.** Association between history of childhood maltreatment and severe COVID-19 outcomes, re-defining the study period before vaccination roll out. **Table S6.** Association between history of childhood maltreatment and severe COVID-19 outcomes, excluding participants registered in Wales as well as re-defining the study period from January 31st, 2020 to July 31st, 2021. **Table S7.** Association between total childhood trauma screener score and COVID-19 outcomes. **Table S8.** Association between any childhood maltreatment and severe COVID-19 outcomes, separating for hospitalization and death due to COVID-19. **Table S9.** Association between history of childhood maltreatment and COVID-19 vaccination.

**Additional file 3.** Regression function for each analysis.

## Data Availability

Data from the UK Biobank (http://www.ukbiobank.ac.uk/) are available to all researchers upon making an application.

## Abbreviations

BMI: Body mass index
CCI: Charlson Comorbidity Index
CM: Childhood maltreatment
CTS: Childhood Trauma Screener
NHS: National Health Service
PC: Principal component
PHE: Public Health England
PHS: Public Health Scotland
PRS: Polygenic risk score
SAIL: Secure Anonymised Information Linkage
TDI: Townsend deprivation index
