# Supplementary material for: History of childhood maltreatment associated with hospitalization or death due to COVID-19: a cohort study
Source: BMC Med. 2024 Aug 7;22:319. doi: 10.1186/s12916-024-03399-8 (PMC11304908; doi:10.1186/s12916-024-03399-8)
Supplement: Supplementary file 1 — Additional file 1: Fig. S1. Study profile. Fig. S2. Spearman rank correlation matrix for different types of childhood maltreatment. Fig. S3. Distribution of cumulative number of childhood maltreatment types. Fig. S4. Principal component on the set of the 10 polygenic risk score for severe COVID-19 outcomes. Fig. S5. Association between history of childhood maltreatment and severe COVID-19 outcomes, by levels of first PRS-PC to severe COVID-19 outcomes. [file 12916_2024_3399_MOESM1_ESM.docx]

Fig. S1 Study profile.


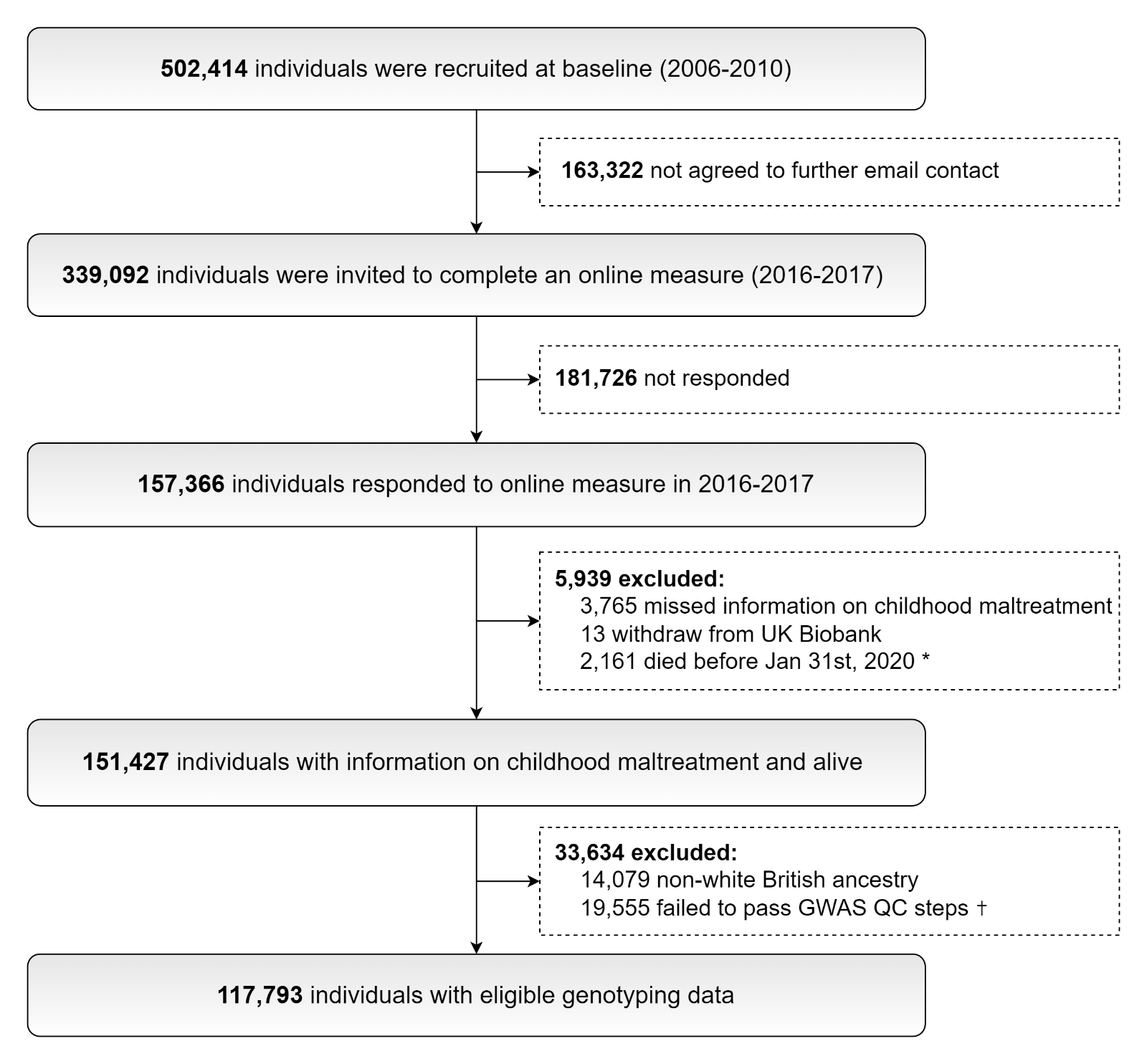


* 1st confirmed COVID-19 cases in the UK

✝ We excluded individuals having genotyping rate <98%, with abnormal heterozygosity level or a kinship coefficient >0.0884.

Fig. S2 Spearman rank correlation matrix for different types of childhood maltreatment.


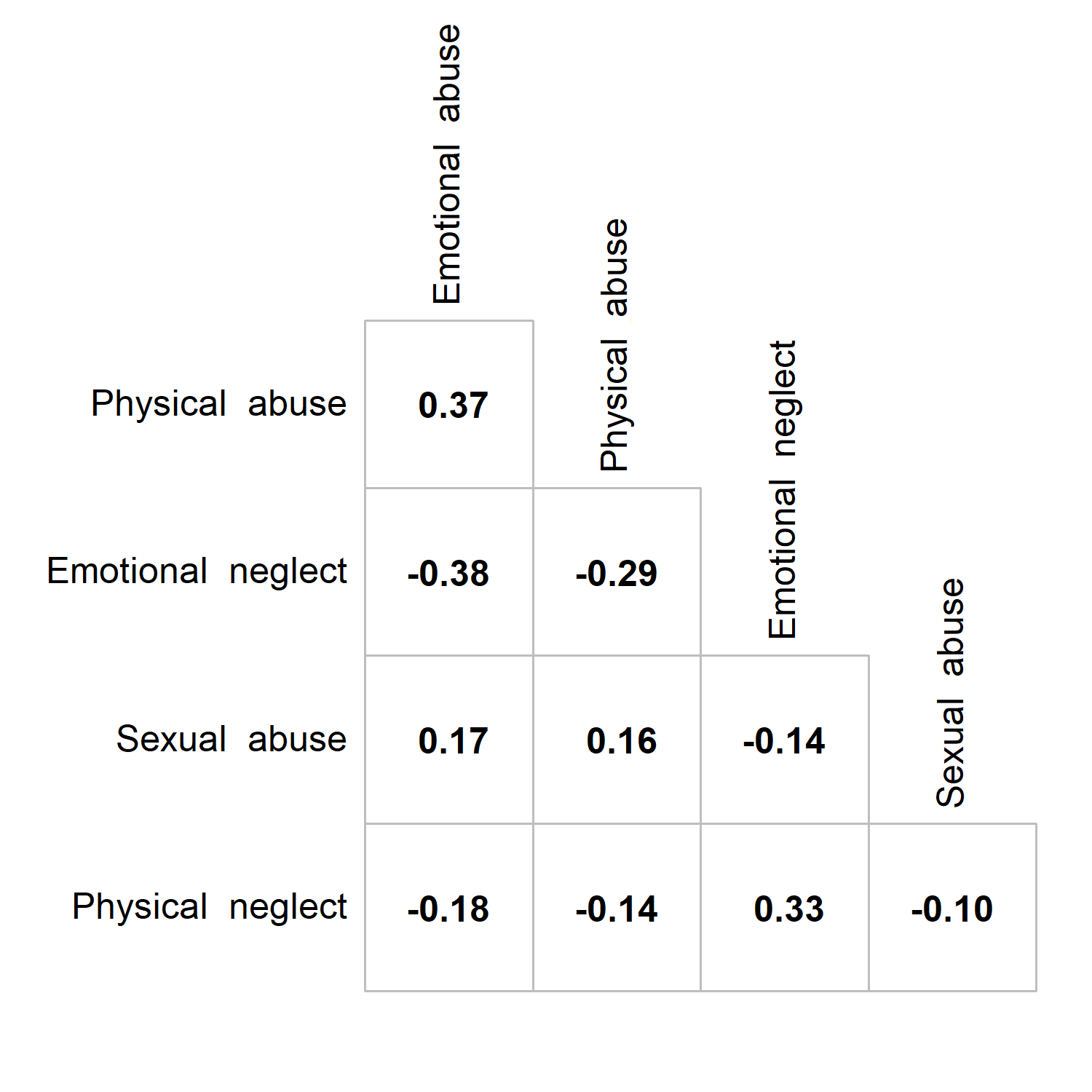


Fig. S3 Distribution of cumulative number of childhood maltreatment types.


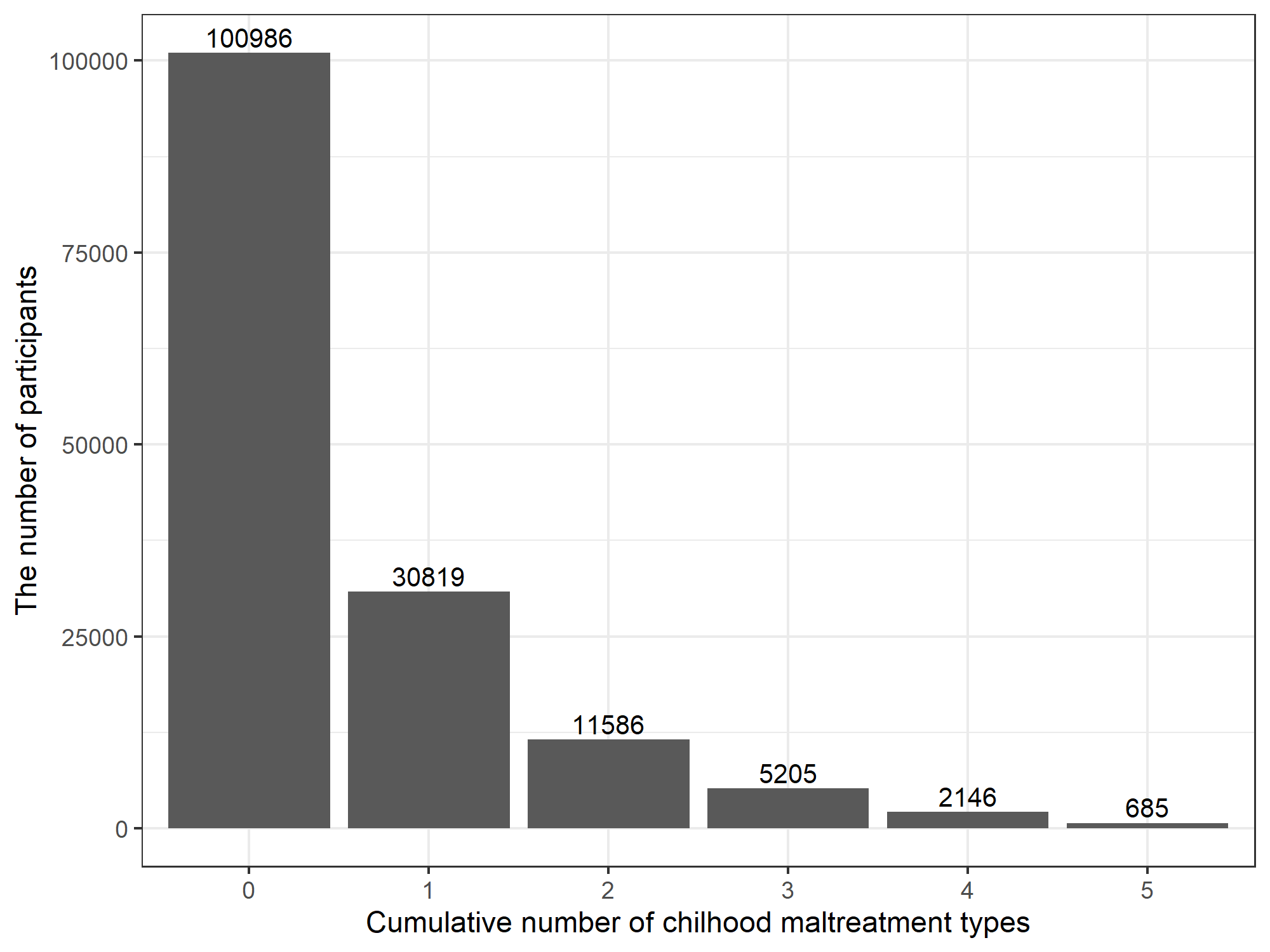


Fig. S4 Principal component (PC) on the set of the 10 polygenic risk score (PRS) for severe COVID-19 outcomes.

| A. Correlation of the PRS based on different p-value thresholds in the studied sample (n=117,793). | | |
| --- | --- | --- |
| 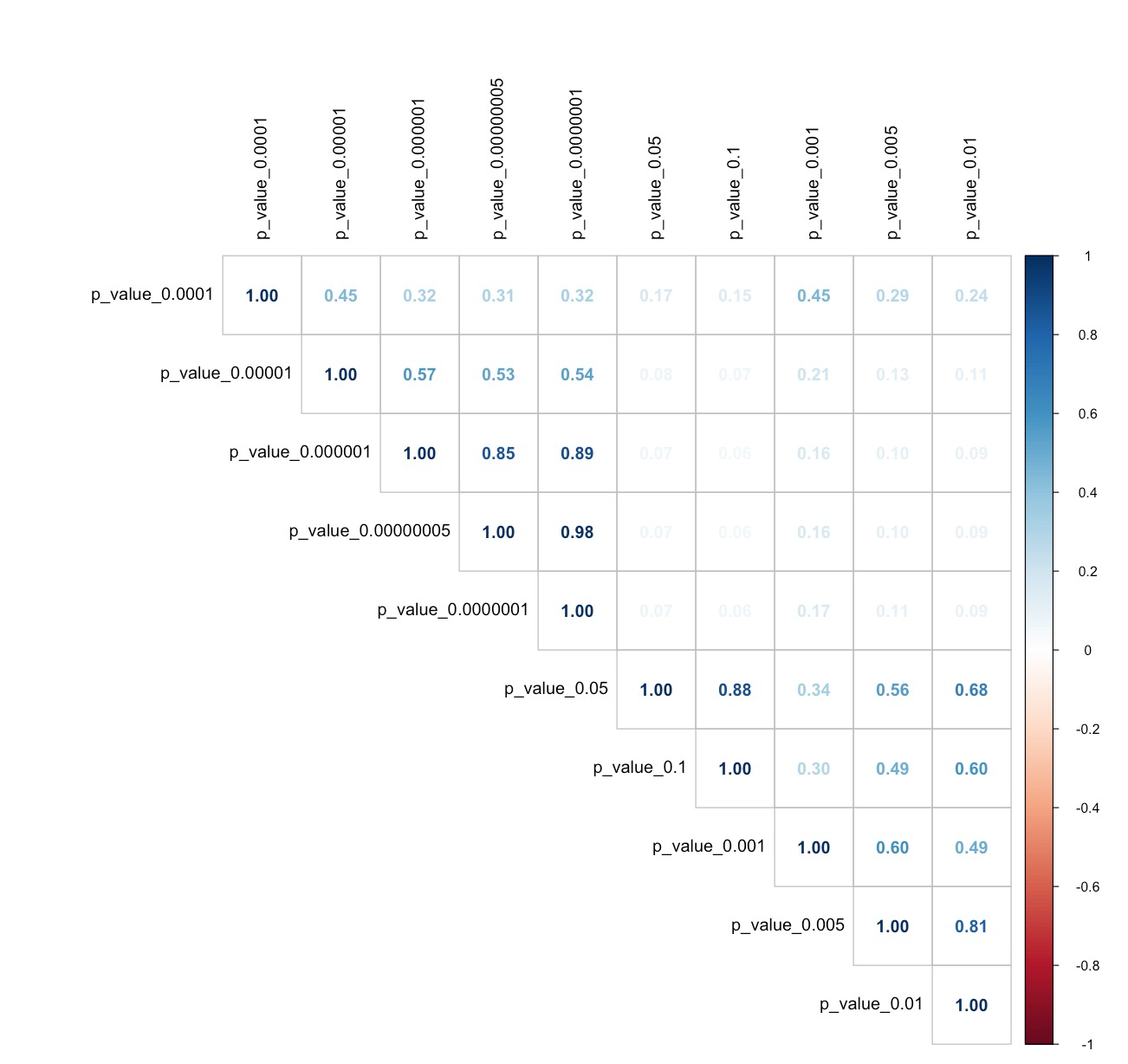 | | |
| B. Percentage explained variance by each PRS-PC. | | C. The loadings in the first PRS-PC at each threshold. |
|  |  | |

Fig. S5 Association between history of childhood maltreatment (CM) and severe COVID-19 outcomes (i.e., hospitalization or death due to COVID-19), by levels of first PRS-PC to severe COVID-19 outcomes.


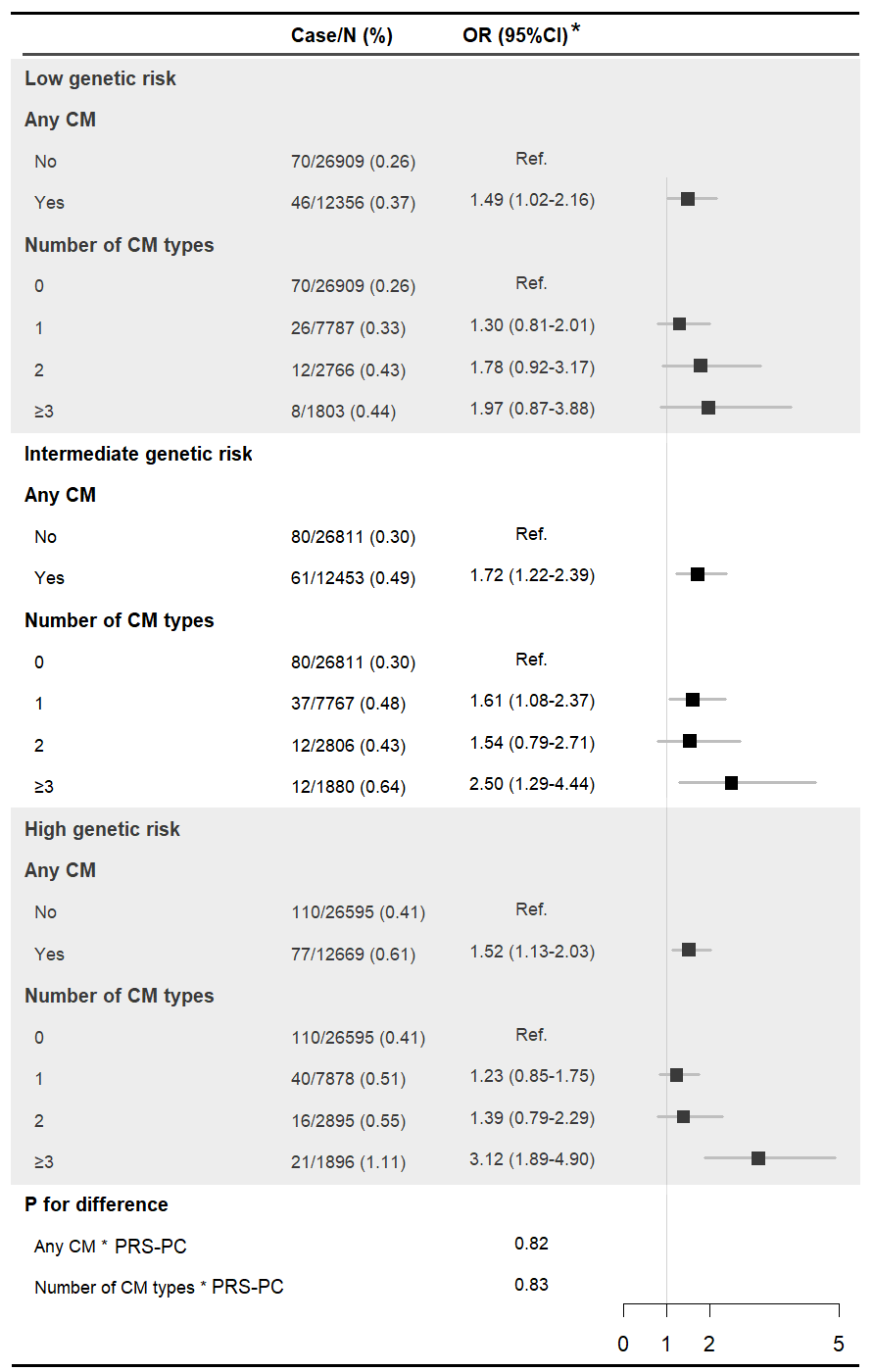


* Adjusted for demographic factors (birth year, sex, ethnicity, and recruitment region).
