# Supplementary material for: History of childhood maltreatment associated with hospitalization or death due to COVID-19: a cohort study
Source: BMC Med. 2024 Aug 7;22:319. doi: 10.1186/s12916-024-03399-8 (PMC11304908; doi:10.1186/s12916-024-03399-8)
Supplement: Supplementary file 3 — Additional file 3. Regression function for each analysis. [file 12916_2024_3399_MOESM3_ESM.docx]

Regression function for each analysis.

**Main analyses (for Table 2):**

- Model 1: Y_hospitalization or death due to COVID-19_ ~ X_any childhood maltreatment_ + birth year+ sex + ethnicity + recruitment region.
- Model 2: Y_hospitalization or death due to COVID-19_ ~ X_any childhood maltreatment_ + birth year+ sex + ethnicity + recruitment region + Townsend deprivation index + college education + annual household income.
- Model 3: Y_hospitalization or death due to COVID-19_ ~ X_any childhood maltreatment_ + birth year+ sex + ethnicity + recruitment region + Townsend deprivation index + college education + annual household income + smoking status + body mass index.
- Model 4: Y_hospitalization or death due to COVID-19_ ~ X_any childhood maltreatment_ + birth year+ sex + ethnicity + recruitment region + Townsend deprivation index + college education + annual household income + smoking status + body mass index + pre-pandemic chronic medical conditions.
- Model 5: Y_hospitalization or death due to COVID-19_ ~ X_any childhood maltreatment_ + birth year+ sex + ethnicity + recruitment region + Townsend deprivation index + college education + annual household income + smoking status + body mass index + pre-pandemic chronic medical conditions + pre-pandemic psychiatric disorders.

We then replaced X_any childhood maltreatment_ with X_number of childhood maltreatment types_, and repeated the above analyses.

**Secondary analyses (for Table 2):**

- Model 1: Y_COVID-19_ _diagnosis_~ X_any childhood maltreatment_ + birth year+ sex + ethnicity + recruitment region.
- Model 2: Y_COVID-19_ _diagnosis_ ~ X_any childhood maltreatment_ + birth year+ sex + ethnicity + recruitment region + Townsend deprivation index + college education + annual household income.
- Model 3: Y_COVID-19_ _diagnosis_ ~ X_any childhood maltreatment_ + birth year+ sex + ethnicity + recruitment region + Townsend deprivation index + college education + annual household income + smoking status + body mass index.
- Model 4: Y_COVID-19_ _diagnosis_ ~ X_any childhood maltreatment_ + birth year+ sex + ethnicity + recruitment region + Townsend deprivation index + college education + annual household income + smoking status + body mass index + pre-pandemic chronic medical conditions.
- Model 5: Y_COVID-19_ _diagnosis_ ~ X_any childhood maltreatment_ + birth year+ sex + ethnicity + recruitment region + Townsend deprivation index + college education + annual household income + smoking status + body mass index + pre-pandemic chronic medical conditions + pre-pandemic psychiatric disorders.

We then replaced X_any childhood maltreatment_ with X_number of childhood maltreatment types_, and repeated the above analyses.

**Secondary analyses (for Additional file 2: Table S9):**

- Model 1: Y_being unvaccinated for COVID-19_ ~ X_any childhood maltreatment_ + birth year+ sex + ethnicity + recruitment region.
- Model 2: Y_being unvaccinated for COVID-19_ ~ X_any childhood maltreatment_ + birth year+ sex + ethnicity + recruitment region + Townsend deprivation index + college education + annual household income.
- Model 3: Y_being unvaccinated for COVID-19_ ~ X_any childhood maltreatment_ + birth year+ sex + ethnicity + recruitment region + Townsend deprivation index + college education + annual household income + smoking status + body mass index.
- Model 4: Y_being unvaccinated for COVID-19_ ~ X_any childhood maltreatment_ + birth year+ sex + ethnicity + recruitment region + Townsend deprivation index + college education + annual household income + smoking status + body mass index + pre-pandemic chronic medical conditions.
- Model 5: Y_being unvaccinated for COVID-19_ ~ X_any childhood maltreatment_ + birth year+ sex + ethnicity + recruitment region + Townsend deprivation index + college education + annual household income + smoking status + body mass index + pre-pandemic chronic medical conditions + pre-pandemic psychiatric disorders.

We then replaced X_any childhood maltreatment_ with X_number of childhood maltreatment types,_ and repeated the above analyses.

**Subgroup analyses (for Fig. 2)**

- Model 1: Y_hospitalization or death due to COVID-19_ ~ X_sexual abuse_ + birth year+ sex + ethnicity + recruitment region.
- Model 2: Y_hospitalization or death due to COVID-19_ ~ X_sexual abuse_ + birth year+ sex + ethnicity + recruitment region + Townsend deprivation index + college education + annual household income.
- Model 3: Y_hospitalization or death due to COVID-19_ ~ X_sexual abuse_ + birth year+ sex + ethnicity + recruitment region + Townsend deprivation index + college education + annual household income + smoking status + body mass index.
- Model 4: Y_hospitalization or death due to COVID-19_ ~ X_sexual abuse_ + birth year+ sex + ethnicity + recruitment region + Townsend deprivation index + college education + annual household income + smoking status + body mass index + pre-pandemic chronic medical conditions.
- Model 5: Y_hospitalization or death due to COVID-19_ ~ X_sexual abuse_ + birth year+ sex + ethnicity + recruitment region + Townsend deprivation index + college education + annual household income + smoking status + body mass index + pre-pandemic chronic medical conditions + pre-pandemic psychiatric disorders.

We the replaced X_sexual abuse_ with X_physical neglect_, X_physical abuse_, X_emotional neglect_, and X_emotional abuse,_ respectively, and then repeated the above analyses.

**Regression-based causal mediation analyses (for Fig. 3)**

- M1: cmest(data, model = "rb",

outcome = Y_hospitalization or death due to COVID-19_,

exposure = X_any childhood maltreatment_,

mediator = c (Townsend deprivation index, college education, annual household income),

basec = c (birth year, sex, ethnicity, recruitment region), # confounder

mreg = list("multinomial","multinomial","multinomial"), yreg = "logistic",

astar = 0, a = 1, mval = list('lower','Yes','Greater than 100,000'), yval=list(1),

estimation = "imputation", inference = "bootstrap", nboot = 10).

- M2: cmest(data, model = "rb",

outcome = Y_hospitalization or death due to COVID-19_,

exposure = X_any childhood maltreatment_,

mediator = c (smoking status, body mass index),

basec = c (birth year, sex, ethnicity, recruitment region), # confounder

mreg = list("multinomial","multinomial"), yreg = "logistic",

astar = 0, a = 1, mval = list('Never','Normal weight'), yval=list(1),

estimation = "imputation", inference = "bootstrap", nboot = 10).

- M3: cmest(data, model = "rb",

outcome = Y_hospitalization or death due to COVID-19_,

exposure = X_any childhood maltreatment_,

mediator = pre-pandemic chronic medical conditions,

basec = c (birth year, sex, ethnicity, recruitment region), # confounder

mreg ="logistic", yreg = "logistic",

astar = 0, a = 1, mval = list('No'), yval=list(1),

estimation = "imputation", inference = "bootstrap", nboot = 10).

- M4: cmest(data, model = "rb",

outcome = Y_hospitalization or death due to COVID-19_,

exposure = X_any childhood maltreatment_,

mediator = pre-pandemic psychiatric disorders,

basec = c (birth year, sex, ethnicity, recruitment region), # confounder

mreg ="logistic", yreg = "logistic",

astar = 0, a = 1, mval = list('No'), yval=list(1),

estimation = "imputation", inference = "bootstrap", nboot = 10).

- M5: cmest(data, model = "rb",

outcome = Y_hospitalization or death due to COVID-19_,

exposure = X_any childhood maltreatment_,

mediator = c (Townsend deprivation index, college education, annual household income, smoking

status, body mass index, pre-pandemic chronic medical conditions, pre-pandemic psychiatric disorders),

basec = c (birth year, sex, ethnicity, recruitment region), # confounder

mreg = list("multinomial", "multinomial","multinomial", "multinomial", "multinomial", "logistic", "logistic"),

yreg = "logistic",

astar = 0, a = 1, mval = list('lower','Yes','Greater than 100,000','Never','Normal weight','No','No'),

yval=list(1),

estimation = "imputation", inference = "bootstrap", nboot = 10).

**Modification analyses (for Fig. 4)**

- Y_hospitalization or death due to COVID-19_ ~ X_any childhood maltreatment_ * Moderator_polygenic risk score (PRS) for COVID-19 hospitalization or death_ + birth year+ sex + ethnicity + recruitment region.
- Y_hospitalization or death due to COVID-19_ ~ X_number of childhood maltreatment types *_ Moderator_polygenic risk score (PRS) for COVID-19 hospitalization or death_ + birth year+ sex + ethnicity + recruitment region.
